# Nanopore-Based Sequencing of the Full-Length Transcriptome of Male and Female Cleavage-Stage Embryos of the Chinese Mitten Crab (*Eriocheir sinensis*)

**DOI:** 10.3390/ijms252212097

**Published:** 2024-11-11

**Authors:** Rui Han, Mengqi Ni, Wentao Lu, Dandan Zhu, Tianyi Feng, Yanan Yang, Zhaoxia Cui

**Affiliations:** 1 School of Marine Sciences, Ningbo University, Ningbo 315020, China; han19990701@163.com (R.H.); 2301130091@nbu.edu.cn (M.N.); lwt970323@163.com (W.L.); zhudd2015@163.com (D.Z.); fengtianyi@nbu.edu.cn (T.F.); yangyanan@nbu.edu.cn (Y.Y.); 2 Laboratory for Marine Biology and Biotechnology, Qingdao National Laboratory for Marine Science and Technology, Qingdao 266071, China

**Keywords:** *Eriocheir sinensis*, full-length transcriptome, male and female cleavage-stage embryos, ribosomal protein genes

## Abstract

The cleavage stage plays a crucial role in embryo development, characterized by a swift surge in cell proliferation alongside the accurate genetic material transmission to offspring. To delve into the characteristics of sex development during the cleavage stage of embryos, we generated the full-length transcriptome of *Eriocheir sinensis* male and female cleavage-stage embryos using Oxford Nanopore Technologies (ONT). Notably, this investigation represents the first sequencing effort distinguishing between genders in *E. sinensis* embryos. In the transcriptome structure analysis, male and female cleavage-stage embryos, while not clustered, exhibited a comparable frequency of alternative splicing (AS) occurrences. We also successfully identified 2875 transcription factors (TFs). The quantitative analysis showed the top 150 genes, in which the highly expressed genes in male embryos predominantly related to protein synthesis and metabolism. Further investigation unveiled 500 differentially expressed genes (DEGs), of which 7 male-biased ribosomal protein genes (RPGs) were particularly noteworthy and further confirmed. These analyses suggest that there may be a more active protein synthesis process in male *E. sinensis* cleavage-stage embryos. Furthermore, among the 2875 identified TFs, we predicted that 18 TFs could regulate the differentially expressed RPGs, with most TFs belonging to the zf-C2H2 and Homeobox families, which are crucial for embryonic development. During the cleavage stage of *E. sinensis*, the differential RPGs between genders were intricately linked to energy metabolism. We proposed that these RPGs exert regulatory effects on gene expression in *E. sinensis*, thereby regulating the difference of development between male and females. Our research sheds light on the developmental mechanisms of *E. sinensis* during the embryo stage and establishes a groundwork for a deeper understanding of sex development in *E. sinensis*. The results also provide comprehensive full-length transcriptome data for future gene expression and genetic studies in *E. sinensis*.

## 1. Introduction

In arthropods, there are differences in early embryonic development between males and females [1]. For instance, research on *Apis mellifera* revealed that a majority of differentially expressed genes (DEGs) in male and female embryos are closely linked to gender development and differentiation within two hours after laying [2]. Similarly, a comparative transcriptome analysis of male and female *Bactrocera jarvisi* embryos during the pre-embryonic stage identified 16 sex-determining genes [3]. Meanwhile, studies on crustaceans, such as *Daphnia magna*, have highlighted notable genetic disparities between male and female embryos [4]. However, research on the relationship between male and female crustacean embryos remains relatively limited.

The cleavage stage serves as the foundation of embryonic development, which is responsible for increasing cell numbers, ensuring proper genetic material distribution, and laying the groundwork for subsequent cell differentiation and organ formation [5,6]. Cell division is the primary biological activity during this stage, involving active protein synthesis and metabolism [7,8]. Research on *Drosophila melanogaster* has demonstrated that impaired ribosome function can significantly impact gene-specific translation efficiency, thereby influencing embryonic development [9]. Meanwhile, embryonic development is regulated by alternative splicing (AS); for instance, different splicing forms of *dUTPase* and *DPTP4E* can modulate embryonic growth [10,11,12]. These AS events play a crucial role in regulating protein synthesis and metabolism during embryonic development. In crustaceans, the cleavage stage not only represents a critical phase of development but also directly influences the reproductive capacity of mature individuals [13,14].

Currently, transcriptome analyses of early developmental stages in male and female crabs focus on the juvenile, which shows morphological differentiation of sex. For instance, in the second juvenile stage of *Portunus trituberculatus*, DEGs between males and females are primarily enriched in pathways associated with DNA replication, folate biosynthesis, and retinol metabolism [15]. Transcriptomic comparisons of *Scylla paramamosain* reveal that DEGs in male and female juveniles are mainly clustered with the steroid biosynthesis pathway [16]. At the third juvenile stage of *Eriocheir sinensis*, differences between males and females are exhibited in metabolic and immune response processes, with no significant correlation with sexual function [17]. Up to now, there has been no comparative study on the identified male and female of crab embryos.

To investigate the early growth and developmental differences between male and female embryos of *E. sinensis* and enhance our comprehension of gender development, we performed nanopore sequencing on cleavage stage male and female embryos of *E. sinensis*. This approach can realize the detection of AS, DEGs, and transcription factors (TFs) based on sequencing results. Our research identified seven up-regulated ribosomal protein genes (RPGs) in male cleavage-stage embryos. Subsequently, predictions suggested that these male-biased RPGs were regulated by 18 TFs associated with embryonic development. A more active protein synthesis process is revealed in male *E. sinensis* cleavage-stage embryos. This research elucidates the developmental mechanisms during this stage, particularly highlighting the key role of RPGs in sex-differentiated development.

## 2. Results

### 2.1. ONT Sequences and Completeness During the Cleavage Stage of E. sinensis

This study employed full-length transcriptome sequencing on the samples. Raw data underwent filtering to obtain clean data, averaging approximately 4.64 GB for females and 4.54 GB for males. Each sample yielded between 2,784,405 and 11,794,902 full-length sequences (Supplementary Table S1). Redundancy was subsequently removed using software, resulting in a sequence length distribution with an N50 of 5314 bp (Supplementary Figure S1). Comparison of all non-redundant transcript sequences with known annotations from the reference genome identified 2629 novel transcripts (Supplementary Figure S2).

### 2.2. Principal Component Analysis and AS Analysis in Transcriptomes

**Figure 1:**
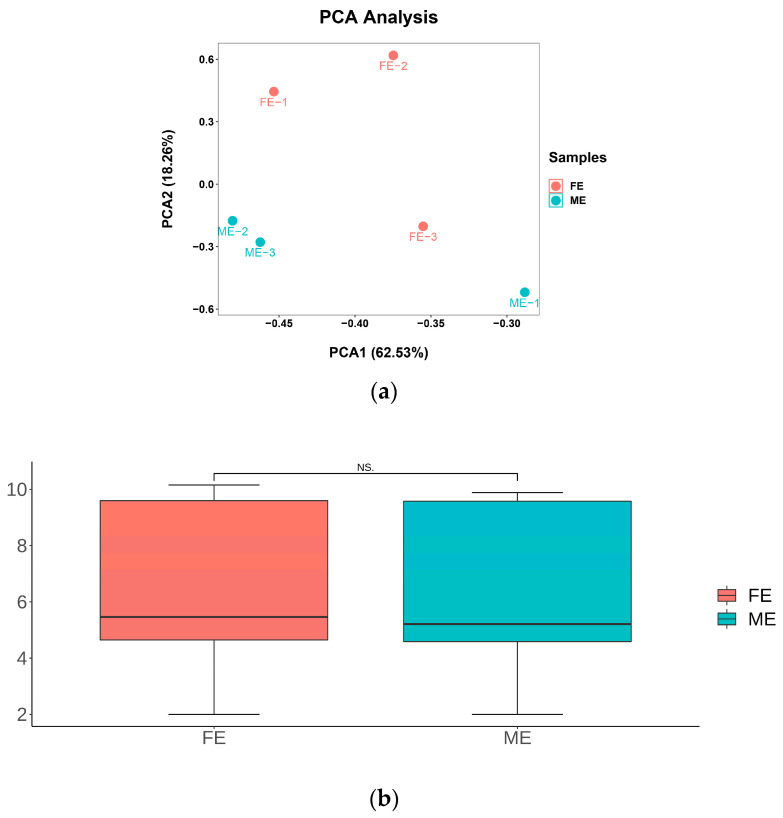
PCA and AS analyses of the female and male of cleavage-stage embryos. (**a**) PCA maps for transcriptomes of FE and ME. (**b**) Number of AS events in male and female cleavage-stage embryos. The x-axis depicts the grouping, while the y-axis represents the number of AS events (processed with log2 logarithms). NS, no significantly difference; FE, female embryos of cleavage stage; ME, male embryos of cleavage stage.

Principal component analysis (PCA) was utilized to assess the differences between male and female samples during the cleavage stage. The results indicated a 62.53% difference between female (FE) and male embryos (ME), with significant dispersion observed within both the male and female groups (Figure 1a). This suggests that *E. sinensis* does not exhibit significant differences between males and females during the cleavage stage. We also conducted an analysis of AS events to examine the patterns separately in male and female cleavage stages. The findings revealed that the incidence and types of AS events were remarkably similar between the male and female transcriptomes, showing no significant differences (Figure 1b and Supplementary Table S4).

### 2.3. Top 150 High Expression Genes in Male and Female Embryos

**Figure 2:**
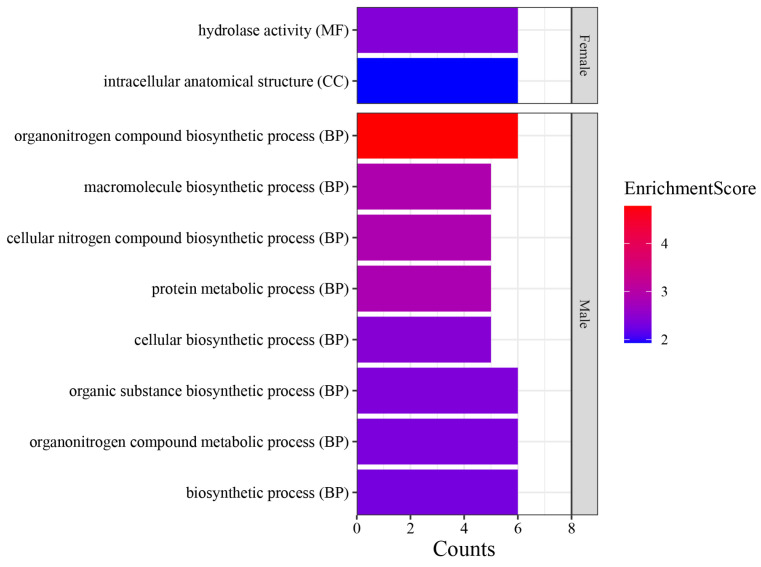
GO enrichment results for the top 150 genes expressed in the female and male of cleavage-stage embryos. The x-axis represents the counts, the y-axis depicts access types, and the color shading indicates the significance level of *p*-values.

We conducted GO enrichment analysis on the 150 most highly expressed genes in female and male cleavage stages (Supplementary Table S5). The results revealed enrichment of two pathways in females: hydrolase activity and intracellular anatomy. In males, eight pathways related to protein synthesis and metabolism were enriched (Figure 2).

### 2.4. Differentially Expressed RPGs Between Male and Female of Cleavage-Stage Embryos

We defined DEGs as genes showing a fold-change coefficient greater than 1 and a *p*-value less than 0.05. A total of 500 DEGs were identified, including 260 up-regulated and 240 down-regulated genes in male compared to female cleavage-stage embryos (Supplementary Table S3). The volcano plot visualizes the differences in the expression levels of DEGs and their statistical significance (Supplementary Figure S3).

**Figure 3:**
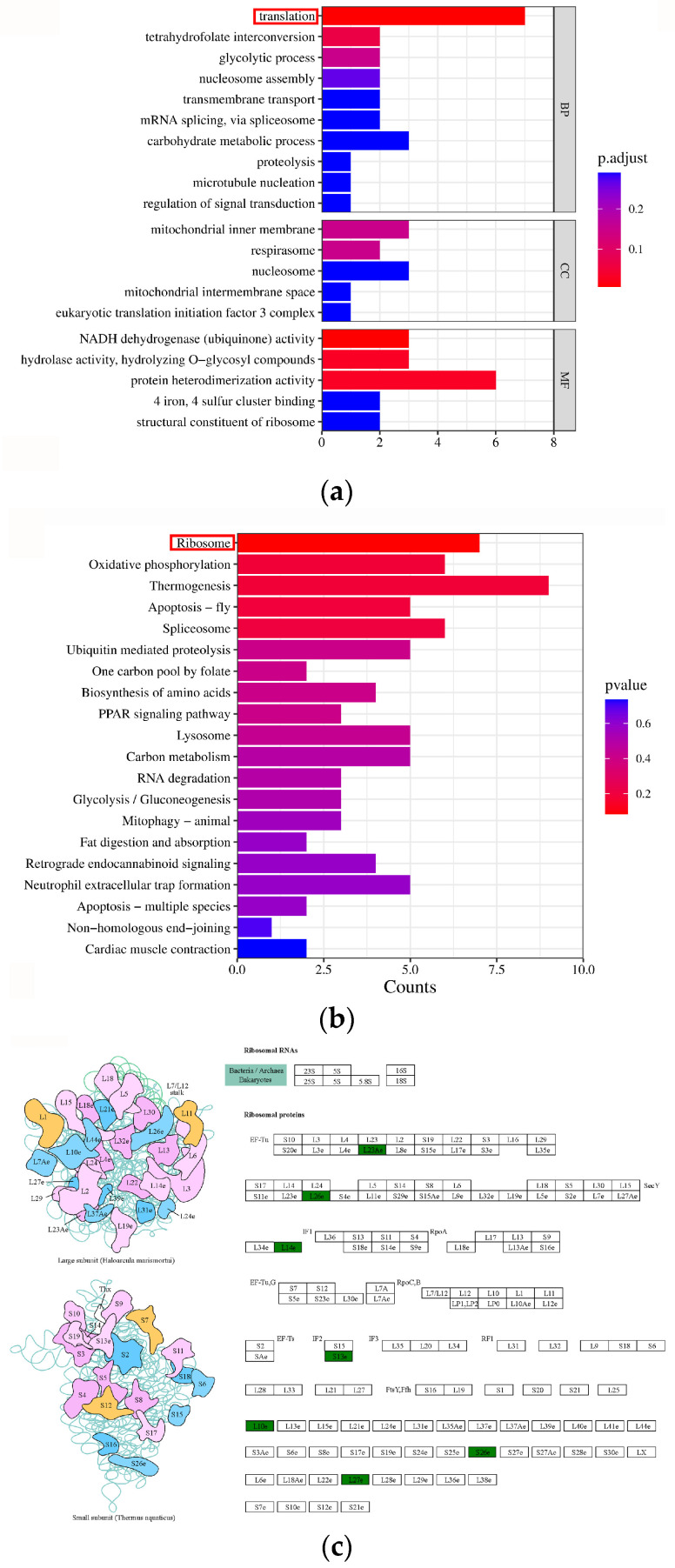

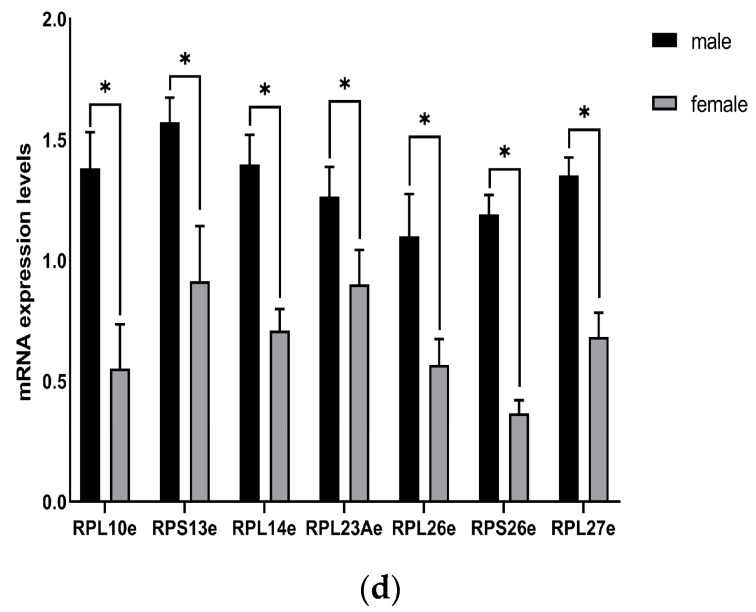
DEG correlation enrichment analysis between male and female of cleavage-stage embryos. (**a**) GO enrichment analysis. (**b**) KEGG enrichment analysis. The horizontal axis represents the number of DEGs. The color represents the *p*-value. The smaller the *p*-value, the more significant the enrichment results. (**c**) Diagram of ribosome structure and constituent subunits. The green blocks represent higher expression of these genes in male cleavage stage embryos. (**d**) Verification of DEGs via qRT-PCR analysis. Each bar represents mean ± SD of triplicate assays. Statistical differences were determined using independent *t*-tests, and asterisks (*) above each bar represent significant differences between male and female cleavage-stage embryos (*p* < 0.05). *RPL10e, Ribosomal protein L10e; RPS13e, Ribosomal protein S13e; RPL14e, Ribosomal protein L14e; RPL23Ae, Ribosomal protein L23Ae; RPL26e, Ribosomal protein L26e; RPS26e, Ribosomal protein S26e; RPL27e, Ribosomal protein L27e*.

Among the 500 DEGs identified, GO enrichment analysis categorized 55 DEGs under biological processes (BP), 17 under cellular components (CC), and 83 under molecular functions (MF). The most significantly enriched pathways for DEGs in MF were NADH dehydrogenase (ubiquinone) activity and hydrolase activity, hydrolyzing O-glycosyl compounds. For BP, the translation emerged as the most significantly enriched pathway, involving seven ribosomal protein genes (RPGs) (Figure 3a).

To identify the significant biochemical, metabolic, and signaling pathways of DEGs during the male and female cleavage stages, KEGG pathway enrichment analysis was performed. A total of 90 DEGs were mapped to established KEGG pathways, with the majority concentrated in ribosomal, oxidative phosphorylation, thermogenesis, apoptosis-fly, and spliceosome pathways (Figure 3b). Notably, among the DEGs we identified, seven RPGs exhibited high transcription levels during the male cleavage stage (Figure 3c). Additionally, these seven DEGs exhibited significant enrichment in the translation pathway within the GO databases.

To assess the accuracy of full-length transcriptome sequencing, we selected seven RPGs for qRT-PCR analysis to further investigate their expression patterns. The analysis revealed significantly higher expression levels of these genes in male cleavage-stage embryos compared to females (*p* < 0.05), which was consistent with the sequencing data (Figure 3d).

### 2.5. Analysis of Ribosomal Genes in E. sinensis

**Figure 4:**
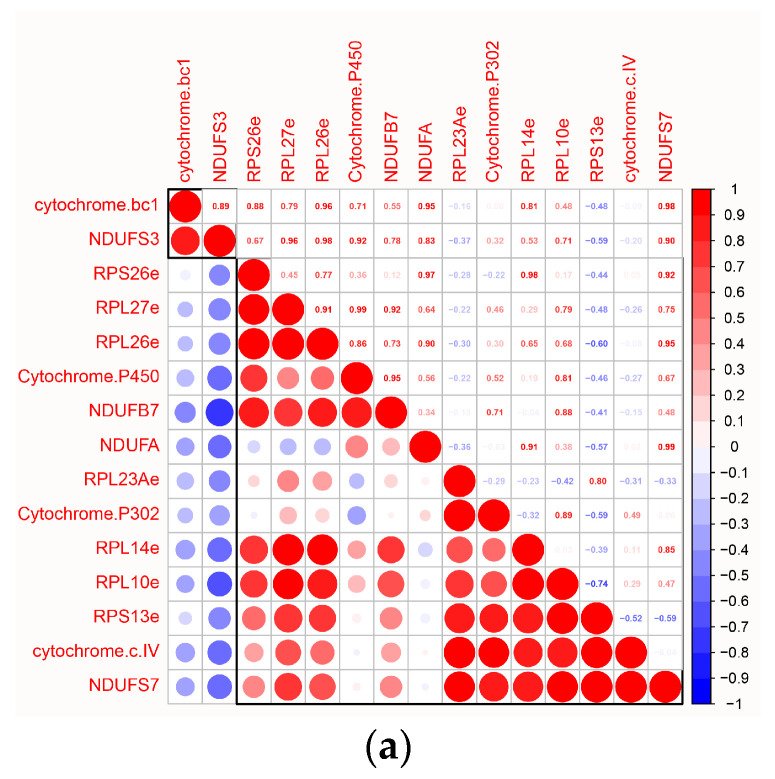

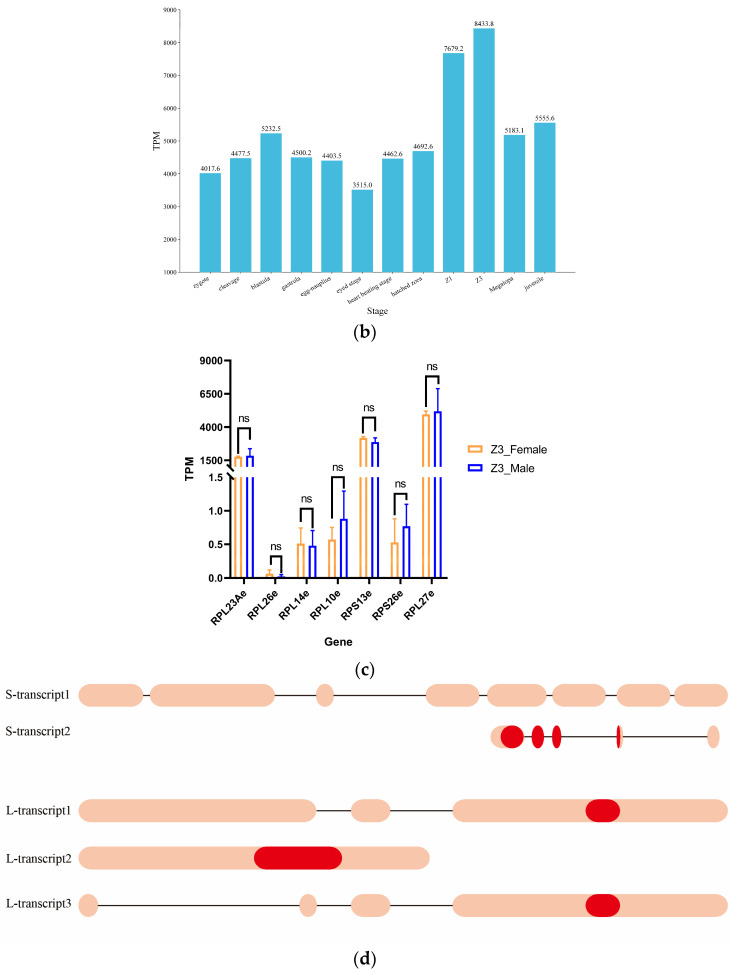
Analysis of ribosomal genes in *E. sinensis*. (**a**) Correlation of differential ribosomal genes in the male and female cleavage stages with energy metabolism-related genes in DEGs. Circle size indicates the degree of relevance: larger circles denote higher relevance; smaller circles denote lower relevance. Color denotes expression in male and female cleavage stages: red indicates expression in both males and females and blue indicates expression only in females or males. *cytochrome. bc1, cytochrome b-c1 complex subunit 9; cytochrome. P450, cytochrome P450 9e2; Cytochrome P302, Cyp302a1; cytochrome.c.IV, cytochrome c oxidase polypeptide VIIc; NDUFS3, NADH dehydrogenase [ubiquinone] iron-sulfur protein 3, mitochondrial; NDUFB7, NADH dehydrogenase [ubiquinone] 1 beta subcomplex subunit 7; NDUFA, NADH dehydrogenase [ubiquinone] 1 alpha subcomplex; NDUFS7, NADH dehydrogenase [ubiquinone] iron-sulfur protein 7, mitochondrial*. (**b**) Ribosomal gene expression during early developmental stages of *E. sinensis*. Z1, Zoea I; Z3, Zoea III. (**c**) Comparison of the expression of the seven RPGs in males and females during Zoea III. Statistical differences were determined using independent *t*-tests, and ns above each bar represent no significant differences between male and female Zoea III. (**d**) Structure comparison of *RPS26e* and *RPL26* transcripts. S-transcripts represent transcripts of RPS26e; L-transcripts represent transcripts of RPL26e; pink squares indicate exons; red frames represent domains.

To explore the effects of ribosomal genes on the development of the cleavage stage of *E. sinensis* embryo, we initially performed correlation analysis of expression between seven RPGs and genes related to energy metabolism among the DEGs. Our findings revealed a strong correlation between RPGs and NADH dehydrogenase-related genes (Figure 4a). In addition, we examined the expression of seven RPGs across various early developmental stages of *E. sinensis*. We observed the highest expression of ribosomal genes during the Zoea III stage but no difference between male and female individuals (Figure 4b,c). We also found alternative splicing events in *RPS26e* and *RPL26e*, while we mapped the transcript structures of the two RPGs (Table 1 and Figure 4d).

### 2.6. TF Regulation on RPGs

**Figure 5:**
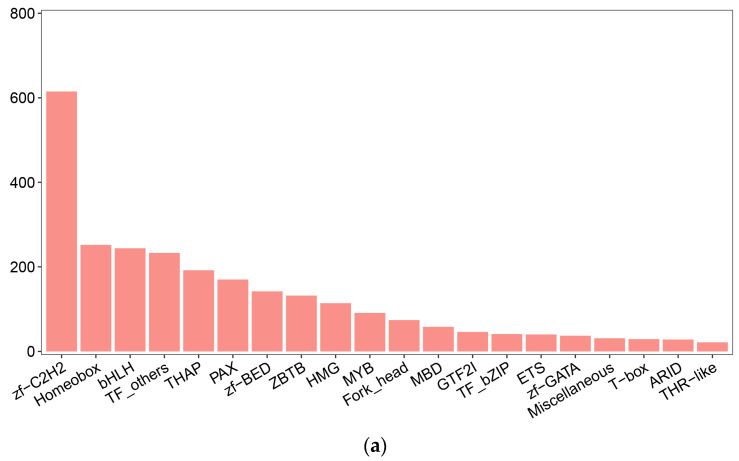

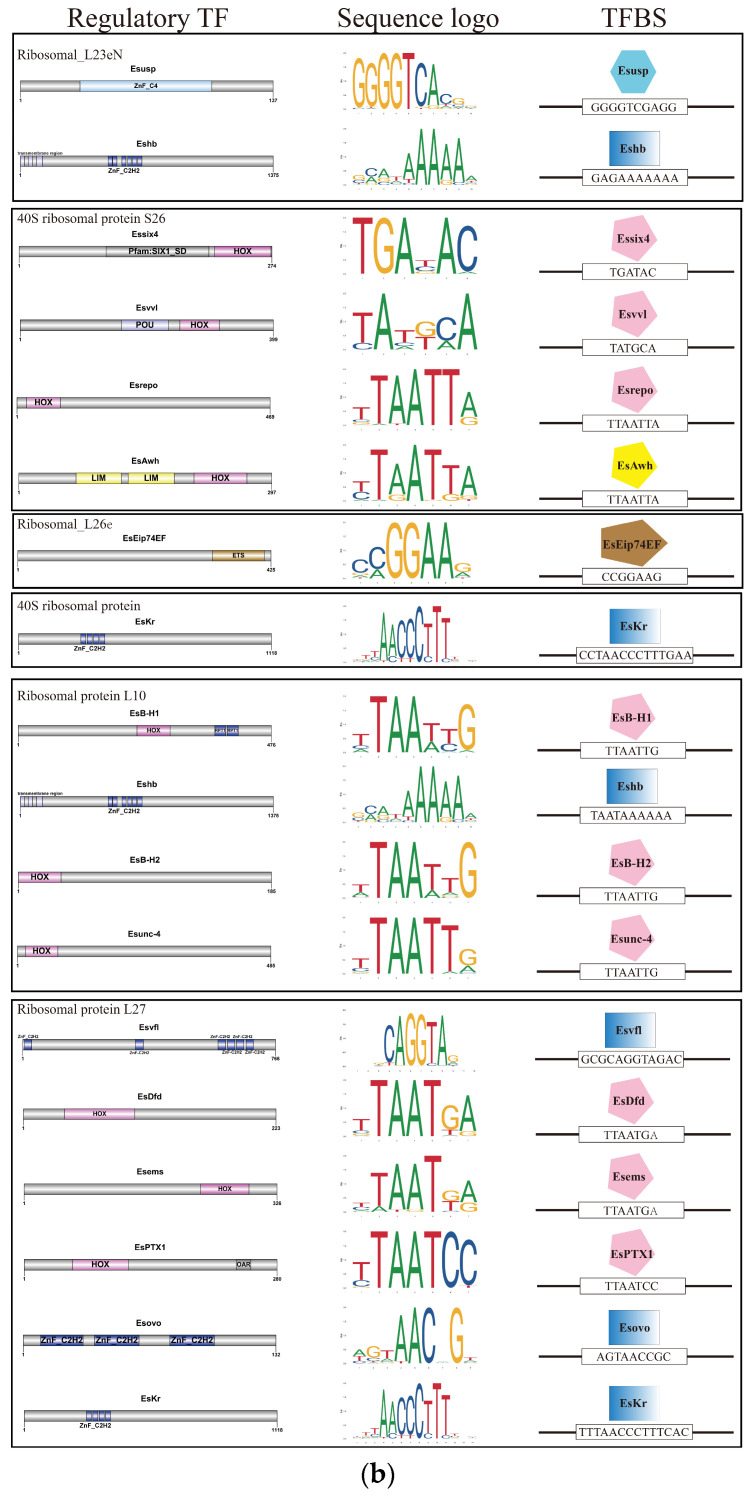
The prediction of TF family. (**a**) Number and top 20 families of predicted TFs. (**b**) Predicted 18 TFs that regulated ribosomal subunits (RPGs). The first column lists TFs and their domains, the second column displays sequence logos, and the third column shows TF binding sites.

We identified a total of 2875 transcription factors (TFs), with the zf-C2H2 family being the most frequently annotated (Figure 5a). To explore the potential transcriptional regulatory mechanisms of seven RPGs, we identified 18 TFs from a pool of 2875 TFs that could potentially affect these genes. These 18 TFs belonged to the zf-C2H2, Homeobox, and ETS (E26 transformation specific) families (Figure 5b). In addition, we identified only one differentially expressed TF, USP, between male and female embryos. USP belongs to the Homeobox family.

## 3. Discussion

The Chinese Mitten Crab, *E. sinensis*, is economically significant in China’s aquaculture. This study used ONT technology to obtain the full-length transcriptome of male and female embryos at the cleavage stage. PCA analysis can provide insights into both inter- and intra-group differences [18]. Based on the PCA results, significant differences are evident not only between males and females during the cleavage stage, but also within the same sexes in this study. It has been reported that the differences in sex development may initiate during larvae stages in some crustaceans. For instance, DEGs between males and females are revealed in Zoea I of *Litopenaeus vannamei* [19]. Similarly, in Zoea II of *P. trituberculatus*, DEGs between males and females primarily enrich growth metabolic pathways [15]. This study represents the first sequencing effort distinguishing between genders in *E. sinensis* embryos.

AS is a vital phenomenon in the development of organisms, and can produce multiple transcript forms [20,21]. In the arthropod *Bombyx mori*, AS plays a role in embryonic development, with the POUM2 gene regulating midgut development [22]. In addition, in the crustacean *Macrobrachium nipponense*, the AS of ecdysteroids contributes to morphogenesis during embryonic development [23]. We identified numerous AS events in male and female cleavage stages of *E. sinensis*, demonstrating its significance in embryonic development. Although there was no difference in AS event numbers between males and females, further research should be focused on the comparative analysis of splicing events in the other sex-identified embryonic stages.

The pathways enriched in the top 150 expressed genes in males are primarily related to protein synthesis and metabolism in this study. This suggests that these proteins are particularly active and crucial during the cleavage stage in male embryos. These pathways are well known for their essential roles in cell division, growth, and development, and energy metabolism of the embryo [24]. In crustacean embryo development, proteins and amino acids are fundamental components in tissue structure formation, comprising a significant portion of the embryo’s composition, such as 63.2% in *Cherax quadricarinatus* embryos [25]. Additionally, besides contributing to tissue structure, proteins may serve as a primary nutritional source for *E. sinensis* embryos [26,27,28]. Therefore, we hypothesize that energy metabolism and consumption pathways are predominantly active processes in male cleavage-stage embryos.

Based on the analysis of DEGs in male and female cleavage stages of embryos, the ribosome stood out as an enrichment pathway. Seven DEGs (RPGs) were identified, namely, ribosomal proteins L23Ae, L27e, L10e, L14e, and L26e, and 40S ribosomal proteins S26e and S13e. Furthermore, *RPS26e* and *RPL26e* produced different transcripts because of alternative splicing. Therefore, alternative splicing not only affects the transcription patterns of genes, but also produces a sex bias between short and long transcripts. This phenomenon has been observed in other genes of *E. sinensis*, such as *Tra2* which is related to sex [29]. Moreover, all of these ribosomal subunit genes exhibited higher expression levels in males. Previous studies have suggested that genes related to ribosomes can show sexually dimorphic expression [30,31], indicating that protein synthesis might be more active in male cleavage-stage embryos compared to females. However, this result contradicts findings in crustacean *D. magna* and insects *Anopheles gambiae* and *D. melanogaster*, where the large and small subunits of the ribosome are more abundantly expressed in females [32,33,34]. This discrepancy may stem from significant differences in the expression of sex-biased genes due to species specificity [35,36].

RPGs play a crucial role in protein synthesis during the early developmental stages of crustaceans [37]. For example, in the Zoea stage of *Neocaridina davidi*, RPGs are involved in cell division [38]. Additionally, RPGs are implicated in various other developmental processes in crustaceans. For example, in *Macrobrachium nipponense*, *RPL24*, *RPS24*, and *RPL10a* contribute to ovarian development and egg formation [39,40,41]. We detected a significant association between RPGs during the cleavage stage of *E. sinensis* and energy metabolism. Specifically, genes such as *cytochrome bc1* and NDUFS3 showed significant negative correlations with all differential RPGs in both male and female cleavage stages. This suggests that RPGs in *E. sinensis* influence energy metabolism levels between male and female cleavage stages by regulating energy metabolism genes.

TFs play an essential role in regulating the transcription of target genes [42,43,44]. To understand the transcriptional patterns of the seven RPGs, we identified TFs based on their TFBS in the embryonic transcriptome. The analysis revealed 18 TFs for the seven RPGs, including members of the zf-C2H2, Homeobox, and ETS families. Notably, the zf-C2H2 and Homeobox families are the most abundant. Previous studies have shown that the zf-C2H2 family may be associated with various physiological processes and developmental regulation [45,46]. In *E. sinensis*, zf-C2H2 is implicated in the developmental transition from the megalopa stage to the first juvenile crab stage [47]. The Homeobox family is recognized for its role in organismal morphological evolution [48], with Hox genes specifically involved in establishing the embryonic body axis in arthropods [49,50]. Notably, the Hox gene *Dfd* regulates body segment development in insects [51,52], suggesting a potential regulatory role in the ribosomal protein L27e in *E. sinensis*. In addition, we identified a differentially expressed TF, USP (ubiquitin-specific peptidase), known for its role in initiating developmental processes [53]. Therefore, we hypothesize that these zf-C2H2 and Homeobox families influence the differential development of male and female embryos through their regulation of RPGs.

## 4. Materials and Methods

### 4.1. Animal and Sample Collection

The embryos of *E. sinensis* were collected from the crab hatcheries in Xinghua, Jiangsu province of China. Selection of embryos was performed at the cleavage stage under an Olympus BX53F2 stereomicroscope [54]. These biological samples were then separated into test tubes and preserved in liquid nitrogen for further use.

### 4.2. DNA/RNA Coextraction from Cleavage Stage of Embryos

DNA/RNA co-extraction was performed on individual embryos during the cleavage stage. The total DNA and RNA of each embryo were extracted using the Tengen DP423 DNA/RNA/Protein Separation Kit (Tengen, Beijing, China). Firstly, the samples were thoroughly homogenized in lysis buffer using the Servicebio KZ-III-F Cryogenic Tissue Mill (Servicebio Technology, Wuhan, China). The samples were then placed on ice for 15 min and subsequently centrifuged at room temperature at 13,000× *g* for 1 min using the Sorvall™ Legend™ Micro 17R microcentrifuge (Thermo Fisher Scientific, Waltham, MA, USA) to separate total DNA with HiBind^®^ DNA mini-columns. The DNA was mixed with absolute ethanol and digested with DNase I for 15 min. Simultaneously, the lysate was centrifuged through a HiBind^®^ RNA mini-column at 13,000× *g* for 1 min to collect total RNA. The total DNA and RNA were then eluted separately into designated tubes and dissolved in 30 µL of DEPC-treated water. The concentration and quality of DNA and total RNA were assessed using a NanoDrop 2000 spectrophotometer (Thermo Fisher Scientific, Waltham, MA, USA). Finally, total RNA was reverse transcribed into cDNA using the CellAmp™ Whole Transcriptome Amplification Kit (TaKaRa, Kyoto, Japan).

### 4.3. Sex Identification of Embryo and Larvae (Zoea III)

The extracted genomic DNA was utilized to distinguish the genetic sex of *E. sinensis* using the female specific primers, based on the polymerase chain reaction (PCR) method as described in our published papers [55,56]. The total volume of the PCR mixture was 25 μL, consisting of 1 μL of diluted cDNA template, 2.5 μL of 10 × PCR buffer, 2 μL of dNTP (10 mM), 0.5 μL of each primer (10 mM), 0.2 μL of rTaq polymerase (TaKaRa, Kyoto, Japan), and 18.3 μL of double-distilled H_2_O. The PCR reaction conditions included an initial denaturation at 95 °C for 3 min, followed by 40 cycles of denaturation at 95 °C for 30 s, annealing at 55 °C for 30 s, and extension at 72 °C for 20 s, and a final extension at 72 °C for 10 min. After running the agarose gel electrophoresis, the male and female could be distinguished with specific bands of about 300 base pairs (bp).

### 4.4. ONT Long Read Processing

A quantity of 500 ng QC-qualified total RNA was collected for each sample, and its volume was adjusted to 9 μL using nuclease-free water. Reverse transcription was performed using Oligo dT as a primer, followed by low-cycle PCR to amplify the full-length cDNA. A final cDNA library was prepared using the PromethION sequencer (Oxford Nanopore Technologies, Oxford, UK) for sequencing over 48–72 h. The raw fastq data were filtered to remove short segments and low-quality reads (less than 50 bp in length, Qscore less than 7), resulting in total pure data. Full-length sequences were aligned with the reference genome (GCA_013436485.1) using minimap2 software (version: 2.17-r941) [57]. After clustering based on the alignment information, consensus sequences were obtained using pinfish software. However, multiple copies of the same transcript might not cluster into the same consensus sequence, resulting in redundant sequences. The obtained consensus sequences were compared with the reference genome (GCA_013436485.1), and the comparison results were processed with StringTie [58] to eliminate redundancy and obtain non-redundant transcript sequences.

### 4.5. Structure Analysis and Gene Functional Annotation

In this study, gffcompare [59] was used to compare transcripts with known genome transcripts. CDS was predicted by TransDecoder. Gene functions were annotated based on Kyoto Encyclopedia of Genes and Genomes (KEGG) [60], Non-Redundant Protein Sequences (NR) [61], Gene Ontology (GO) [62], Protein Families (Pfam) [63], Eukaryotic Homologous Group/Cluster of Homologous Groups (KOG/COG) [64,65], and Reviewed Protein Sequence Database (Swiss-Prot) [66].

### 4.6. Quantitative Genes and DEGs Analysis

The expression levels were normalized across different genes using TPM (Transcripts Per Kilobase Million) as the metric. Gene expression was quantitatively analyzed using Salmon [67]. Principle component analysis (PCA) was performed using OmicStudio tools available at https://www.omicstudio.cn/tool (accessed on 18 July 2024). The differential expression analysis was conducted using read count data obtained from gene expression quantification in each sample. Differential expression analysis was conducted using DESeq2 software (version: 1.26.0) [68], with a screening threshold set at a *p*-value < 0.05 and |log2FoldChange| > 1. Genes meeting these criteria were classified as DEGs. Meanwhile, correlation analysis between genes was performed using the corplot model from the R package.

### 4.7. Correlation Analysis of Seven RPGs at Cleavage Stage and Expression in Early Developmental Stages

Correlation analysis was performed using the OmicStudio tools at https://www.omicstudio.cn/tool/62 (accessed on 28 July 2024). Transcriptome data for the early developmental stages of *E. sinensis* (including zygote (SRX1818354), blastula stage (SRX10054185), gastrula stage (SRX10054186), egg-nauplius stage (SRX1818359), eyed stage (SRX1818360), heart beating stage (SRX1818361), Zoea I stage (SRX7412182, SRX7412183, SRX7412184, SRX7412185, SRX7412186, SRX7412187), Zoea III stage (SRX15553341, SRX15553342, SRX15553343, SRX15553344, SRX15553345, SRX15553346), megalopa stage (SRX7026500, SRX7026431), and juvenile stage (SRX13837311, SRX13837312, SRX13837311)) were downloaded from NCBI. Seven RPGs were identified based on annotation results from the Nr database, enabling observation of ribosomal gene expression across all developmental stages.

### 4.8. Validation of DEGs by Quantitative Real-Time PCR

DEGs were verified using fluorescence quantitative real-time polymerase chain reaction (qRT-PCR). Total RNA was extracted from cleavage-stage embryos of both male and female *E. sinensis* and reverse transcribed using the PrimeScript TM RT kit (TaKaRa, Shiga, Japan) following the manufacturer’s protocol. QRT-PCR was conducted using the 7500 real-time PCR system (Applied Biosystems, Carlsbad, CA, USA) and TB Green^®^ Premix DimerEraser™ (TaKaRa, Japan) in a total reaction volume of 20 μL, including 10 μL of ROX Reference Dye II (TaKaRa, Japan), 0.5 μL of each primer (10 mM), 2 μL of diluted cDNA, and 7 μL of ddH_2_O. The reaction protocol involved an initial denaturation step at 95 °C for 2 min, followed by 40 cycles of 95 °C for 15 s, 60 °C for 30 s, and 72 °C for 30 s. Selected DEGs included *ribosomal protein L10e (RPL10e), ribosomal protein S13e (RPS13e), ribosomal protein L14e (RPL14e), ribosomal protein L23Ae (RPL23Ae), ribosomal protein L26e (RPL26e), ribosomal protein S26e (RPS26e), and ribosomal protein L27e (RPL27e)*. Gene-specific primers were designed based on Illumina sequencing data using Primer 5.0 software, with β-actin serving as an internal amplification control (Table 2).

### 4.9. Alternative Splicing and Transcription Factors Analysis

AS events were identified in each sample using suppa2 software (version: 2.3) [69]. The original annotated transcript structure was refined by extending the upstream region of the 5′ end using gffcompare. TFs for *E. sinensis* were identified using the Animal Transcription Factor Database [70]. We also performed the predictive analysis of transcription factor binding sites (TFBSs) on gene promoter sequences using https://guolab.wchscu.cn/AnimalTFDB4/#/TFBS_Predict (accessed on 1 April 2024).

### 4.10. Statistical Analysis

Data were analyzed using GraphPad Prism 9.3 software with one-way ANOVA. Results are presented as mean ± SD values, and statistical significance was set at *p* < 0.05.

## 5. Conclusions

Our study aims to complement the embryonic transcriptome data of identified male and female *E. sinensis* using third-generation sequencing technology. We discovered 500 DEGs from the cleavage stage between males and females. Seven male-biased RPGs suggested that protein synthesis may be more active in male *E. sinensis* cleavage-stage embryos. Among the 2875 predicted TFs, we identified 18 that may regulate the seven male-biased RPGs. We propose that TFs from the zf-C2H2 and Homeobox families influence differential embryo development by regulating ribosomal subunits. Our findings hypothesize that RPGs may lead to differences in energy metabolism and growth and development in male and female cleavage-stage embryos by USP regulation. This study refines the full-length transcripts of the *E. sinensis* embryo and highlights RPGs as potential regulators of male and female cleavage-stage embryo growth.

## Figures and Tables

**Table 1:** Differently expressed ribosomal genes with alternative splicing event.

Gene	Number of AS Events	Transcripts
Ribosomal protein S26e (*RPS26e*)	5 (including two A3 events, two A5 events and one RI event)	S-transcript1 (*p* = 0.669)
		S-transcript2 (*p* = 0.028)
Ribosomal protein L26e (*RPL26e*)	2 (including one A5 event and one RI event)	L-transcript1 (Almost no expression in males and females)
		L-transcript2 (*p* = 0.610)
		L-transcript3 (*p* = 0.026)

A3 represents alternative 3′ splice site; A5 represents alternative 5′ splice site; RI represents retained intron; *p*-value represents the difference in transcript expression in males and females. The significant difference between male and female is shown if the value of *p* is <0.05.

**Table 2:** The summary of primers in this study.

Gene	Primer Sequence (5′-3′)	Experiments
*RPL10e-F*	CCTAAGAGTCGGTTCTGCCG	qRT-PCR
*RPL10e-R*	CCTCACCTTGTTGGCACAGA	qRT-PCR
*RPS13e-F*	CATGGTGAAGGGCCAGGATT	qRT-PCR
*RPS13e-R*	CAGGGCTTTCGTAGGGGTTT	qRT-PCR
*RPL14e-F*	CTGACCTGACGGAACACTGG	qRT-PCR
*RPL14e-R*	TGGGGTGTTTGGGCATTGAT	qRT-PCR
*RP L23Ae-F*	GCTAGGAAGGCGTGATTGGT	qRT-PCR
*RP L23Ae-R*	CAAGTTTGTCGTTGTGCCGT	qRT-PCR
*RPL26e-F*	CTTCCAAGGACGCCTCAGTT	qRT-PCR
*RPL26e-R*	CCTTTCTTCCTCTGCGGGTT	qRT-PCR
*RPL27e-F*	ACCTTCGCAATTCTTCCCGT	qRT-PCR
*RPL27e-R*	AGCGTAGTGGTTTGGTGGTT	qRT-PCR
*RPS26e-F*	CATCACACCCGAGGACCAAA	qRT-PCR
*RPS26e-R*	TTGCCTCACTCGACAAACGA	qRT-PCR
*β-actin-F*	GCATCCACGAGACCACTTACA	qRT-PCR
*β-actin-R*	CTCCTGCTTGCTGATCCACATC	qRT-PCR

## Data Availability

Data used in this article have been submitted to the NCBI SRA database (Bioproject: PRJNA1100768).

## Supplementary Materials

The following supporting information can be downloaded at: https://www.mdpi.com/article/10.3390/ijms252212097/s1.
