# Supplementary material for: Nanopore-Based Sequencing of the Full-Length Transcriptome of Male and Female Cleavage-Stage Embryos of the Chinese Mitten Crab (Eriocheir sinensis)
Source: Int J Mol Sci. 2024 Nov 11;25(22):12097. doi: 10.3390/ijms252212097 (PMC11594077; doi:10.3390/ijms252212097)
Supplement: Supplementary file 1 [file ijms-25-12097-s001.zip › Supplementary.pdf]

Supplementary Table

Supplementary Table S1. Sequencing data volume information statistics

Sample Name	Seq Num	Mean Length(bp)	N50(bp)	Max Length(bp)
FE-1	4,618,381	100	207	39,646
FE-2	2,784,405	93	154	12,929
FE-3	11,794,902	115	266	73,071
ME-1	6,372,839	108	253	64,733
ME-2	9,288,118	110	255	145,393
ME-3	7,275,239	117	319	46,122

Supplementary Table S2. Novel transcript function annotation results in statistics

Item	Count	Percentage
All	2,629	100.00%
Annotation	226	8.60%
UniProt	211	8.03%
Pfam	173	6.58%
GO	158	6.01%
KEGG	128	4.87%
Pathway	98	3.73%
KOG	2	0.08%
Nr	206	7.84%

Supplementary Table S4. Alternative splicing type statistics

AS type	FE-1	FE-2	FE-3	ME-1	ME-2	ME-3
Alternative 3' splice site (A3)	941	775	1,138	896	946	889
Alternative 5' splice site (A3)	842	733	1,017	858	847	762
Alternative First exon (AF)	44	32	52	40	32	37
Alternative Last exon (AL)	26	17	35	25	25	32
Mutually exclusive exon (MX)	7	4	4	6	4	5
Retained intron (RI)	706	559	857	712	709	661
Skipping exon (SE)	25	22	28	16	17	24

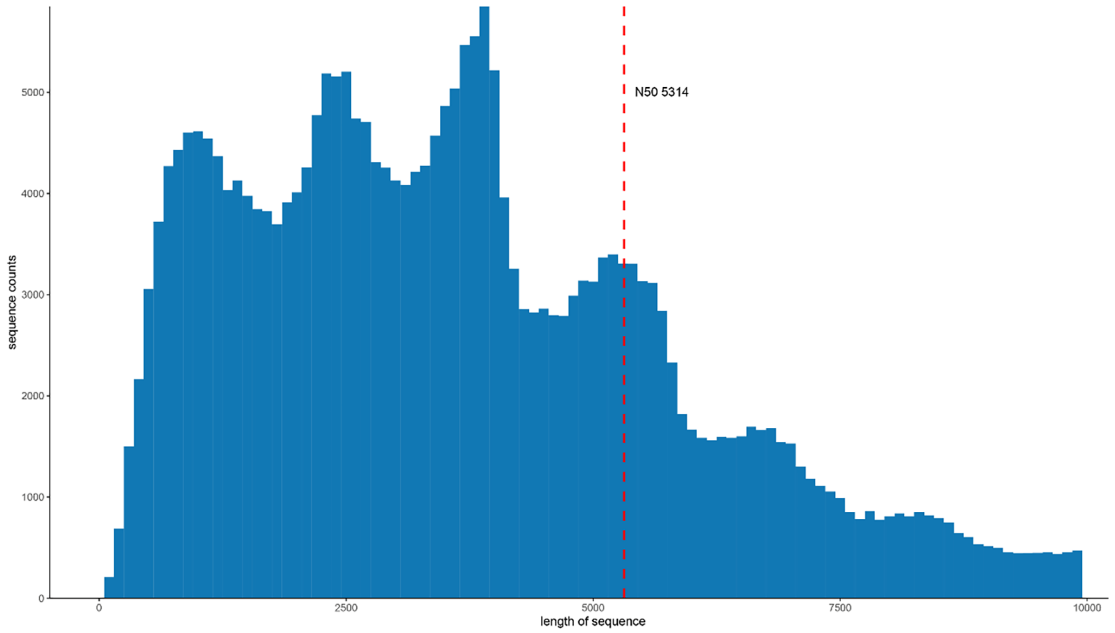

Supplementary figure S1. Distribution of coherent sequence lengths after redundancy

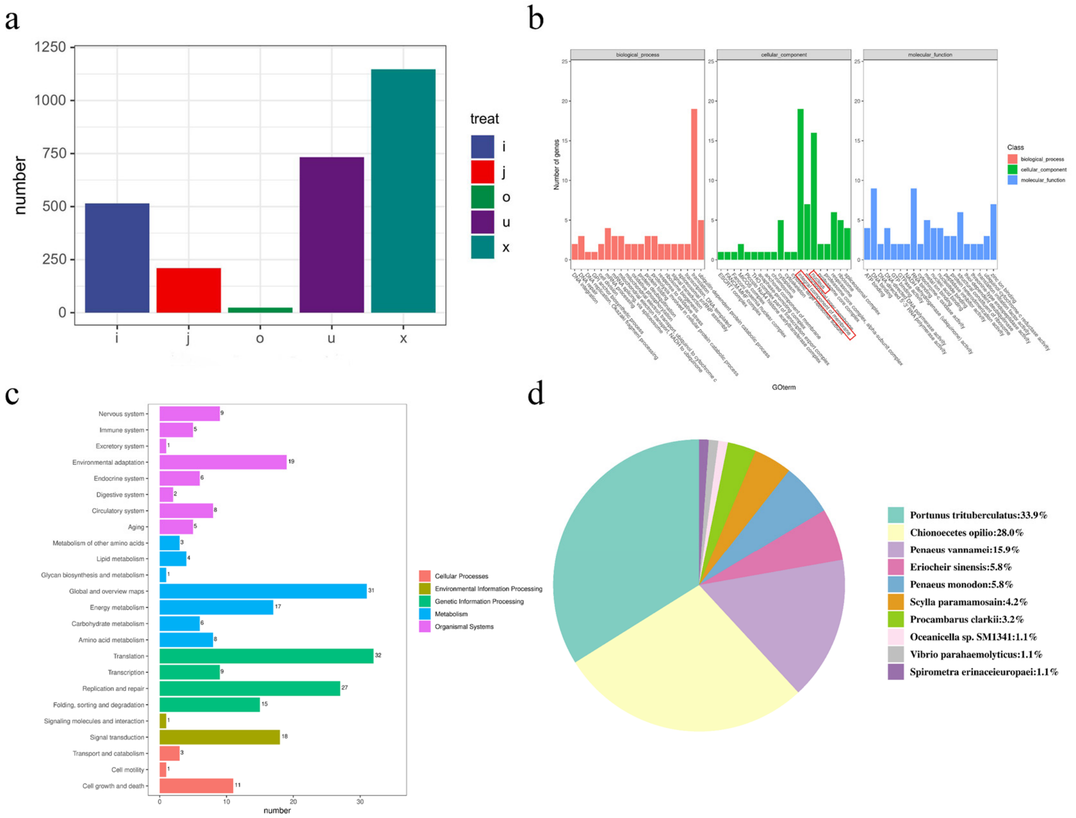

Supplementary figure S2. Analysis of novel transcripts

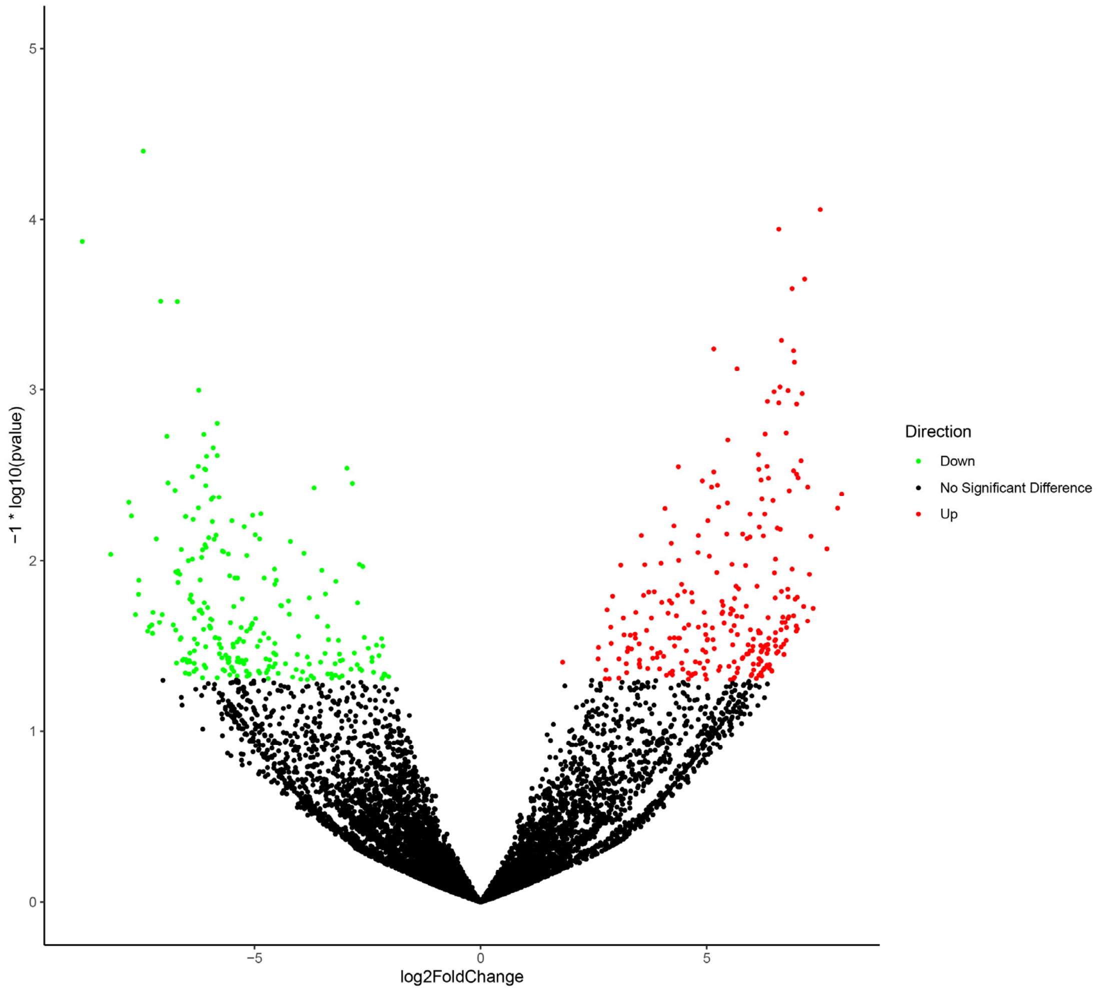

Supplementary figure S3. Volcano plot of DEGs
